# Do AML patients with DNMT3A exon 23 mutations benefit from idarubicin as compared to daunorubicin? A single center experience

**DOI:** 10.18632/oncotarget.347

**Published:** 2011-11-09

**Authors:** Olivier LaRochelle, Sarah Bertoli, François Vergez, Jean-Emmanuel Sarry, Véronique Mansat-De Mas, Sophie Dobbelstein, Nicole Dastugue, Anne-Claire Strzelecki, Cindy Cavelier, Laurent Creancier, Arnaud Pillon, Anna Kruczynski, Cécile Demur, Audrey Sarry, Françoise Huguet, Anne Huynh, Christian Récher, Eric Delabesse

**Affiliations:** ^1^ Laboratoire d'Hématologie, CHU de Toulouse, Hôpital Purpan; ^2^ Service d'Hématologie, CHU de Toulouse, Hôpital Purpan, 31059 Toulouse, France; ^3^ INSERM UMR1037-Cancer Research Center of Toulouse, CNRS ERL 5294, Pavillon Lefebvre BP3028, CHU Purpan, 31024 Toulouse, France; ^4^ Université Toulouse III Paul Sabatier, Toulouse, France; ^5^ Centre de Recherche et Développement Pierre Fabre, 3 avenue Hubert Curien, BP 13562, 31035 Toulouse Cedex 1, France

**Keywords:** DNMT3A, acute leukemia, idarubicin, NOD/SCID, xenograft

## Abstract

Mutations in *DNMT3A* encoding DNA methyltransferase 3A were recently described in patients with acute myeloid leukemia. To assess their prognostic significance, we determined the mutational status of *DNMT3A* exon 23 in 288 patients with AML excluding acute promyelocytic leukemia, aged from 18 to 65 years and treated in Toulouse University Hospital. A mutation was detected in 39 patients (13.5%). All *DNMT3A* exon 23+ patients had intermediate-risk cytogenetics. Mutations significantly correlated with a higher WBC count (p<0.001), *NPM1* (p<0.001) and *FLT3-ITD* mutations (p=0.027). *DNMT3A* mutations were conserved through xenotransplantation in immunodeficient mice. No difference in outcome between *DNMT3A* exon 23+ and *DNMT3A* exon 23- patients was found even if the results were stratified by *NPM1* or *FLT3-ITD* status. However, *DNMT3A* exon 23+ patients had better median DFS (not reached *vs* 11.6 months, p=0.009) and OS (not reached *vs* 14.3 months, p=0.005) as compared to *DNMT3A* exon 23- patients when treated with idarubicin, whereas patients treated with daunorubicin had similar outcome regardless the *DNMT3A* status. This study shows that *DNMT3A* mutations have no impact on outcome but could be a predictive factor for response to idarubicin and thus, could have a direct influence in the way AML patients should be managed.

## INTRODUCTION

Acute myeloid leukemia (AML), most widespread acute leukemia in adults, is characterized by clonal proliferation of oncogenic event-transformed hematopoietic stem cells or progenitors. Despite a high rate of complete remission after treatment with intensive chemotherapy combining cytarabine and anthracyclines in schemas that have little changed during the past 30 years, relapse rates are very high, resulting in a poor outcome in most cases with 5-year overall survival of 40% in younger adults and only 10-15% in elderly patients [1]. Nevertheless, therapeutic results drastically vary regarding the main prognostic factors including age, performance status, cytogenetic and molecular abnormalities. Indeed, the knowledge of molecular basis of AML has considerably increased in the past few years mostly through the identification of recurrent mutational events occurring in a substantial number of patients [2].

The leukemic clone emerges from normal hematopoietic stem cells or more mature myeloid progenitors after acquisition of gene mutations affecting cell differentiation and self-renewal (such as the fusion genes *PML-RARA, AML1-ETO, CBFb-MYH11* or point mutations targeting the functions of *CEBPA, RUNX1, MLL* or *NPM1*), cell signaling (*FLT3, RAS* or *KIT*), epigenetic machinery (*TET2*) and cell metabolism (*IDH1* or *IDH2*) [3-4]. These mutations particularly occur in AML without chromosomal abnormalities detected (normal karyotypes) and represent a major issue in the clinical management of patients since they could provide targets for both therapy (i.e., tyrosine kinase inhibitors in *FLT3-ITD* positive AML) and molecular monitoring of residual disease. However, the major impact of these mutations resides in the stratification of consolidation therapy once complete response is achieved following induction chemotherapy. It is now generally accepted that patients with normal karyotype and favorable genotype (i.e., *NPM1* or *CEBPA* mutations without *FLT3-ITD* mutations) are no longer referred to allogeneic hematopoietic stem-cell transplantation (HSCT) in first complete response since their outcome with chemotherapy alone is similar to those receiving HSCT [5]. In addition, patients with an unfavorable genotype are more readily directed to allograft from unrelated donors or cord blood units since their prognosis with chemotherapy alone is dismal. Thus, *FLT3-ITD, CEBPA, NPM1* mutations are now part of the initial work up of all AML patients with intermediate cytogenetic risk who undergo intensive treatments [6]. However, newly discovered mutations such as isocitrate dehydrogenase 1 and 2 (*IDH1/2*) mutations have been already proposed to refine this molecular stratification although their prognostic impact remains to be definitely established [7-8].

Even more recently, by sequencing the genome of leukemic cells from a patient with AML, Ley et al have detected somatic mutations in the gene of a DNA methyltransferase (MTase), *DNMT3A* [9]. *DNMT3A* is a member of the DNA MTases family including *DNMT1, DNMT2, DNMT3A* and *DNMT3B* that are involved in the methylation of CpG islands [10]. The hypermethylation of CpG islands that contributes to the downregulation of gene expression, and notably of tumor suppressor genes, is also a hallmark of AML [11]. In a series of 281 de novo AML, *DNMT3A* mutations were found in 22% of cases, clustering in intermediate-risk cytogenetic group and associated with a poor outcome. Most of the *DNMT3A* mutations (60%) found in AML samples were missense mutations localized on the R882 amino acid of the MTase domain. It is noteworthy that R882 mutations were significantly associated with a high white blood cell count as compared with other *DNMT3A* mutations. The aim of our study was to confirm the prognostic impact of R882 *DNMT3A* mutations in a series of 288 AML patients treated in our institution and to evaluate its screening usefulness in the medical diagnosis process, in a similar way to KIT exon 17 screening.

## PATIENTS AND METHODS

### Patients and treatments

**Figure 1:**
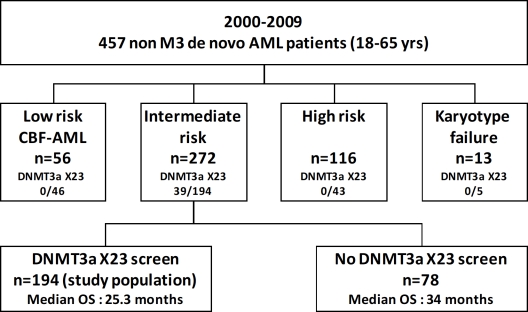
Flow chart of AML patients treated by intensive chemotherapy between 2000 and 2009 From 2000 to 2009, 457 consecutive patients with de novo AML were treated by intensive chemotherapy. Patients with acute promyelocytic leukemia or with secondary AML were excluded from this study.

From 2000 to 2009, 457 consecutive patients (18-65 years) with untreated *de novo* AML (excluding acute promyelocytic leukemia and secondary AML) were admitted for intensive chemotherapy induction at the Hematology department of Toulouse University hospital. In this cohort of patients, 288 samples were available for genetic screening. Since *DNMT3A* mutations were only observed in the intermediate-cytogenetic risk group, the therapeutic outcome was assessed in this population (n=194). Figure 1 shows the flow chart of all AML patients in this period of time. From this cohort of 194 intermediate-risk patients, those ≤ 60 years (n=156) received either daunorubicin (dnr) (60 mg/m2, d1-3) (n=112) or idarubicin (ida) (8 mg/m2, d1-5) (n=44) with cytarabine (AraC) (200 mg/m2, d1-7). A second course (ida, 8mg/m2 or dnr, 35mg/m2 d17-18 and AraC, 1g/m2/12h d17-20) was delivered if more than 5% marrow blasts persisted on d15 [12]. Patients were treated according to institutional guidelines using daunorubicin as main anthracycline while some of them received idarubicin as part of a randomized clinical trial [12]. Patients in complete response (CR) with HLA-identical sibling were planned to receive allo-HSCT: (i) after a myeloablative conditioning regimen consisting of oral busulfan (16 mg/kg over 4 days) and cyclophosphamide (120 mg/kg over 2 days) for patients less than 51 years; (ii) after one course of intensive consolidation described below and reduced intensity conditioning, (oral busulfan, 8 mg/kg over 2 days, fludarabine, 120 mg/m2 over 4 days, rabbit anti-thymocyte globulins 2,5 mg/kg on d-4 and d-3) for patients between 51 and 60 years. Patients with no HLA-identical sibling received a consolidation regimen (ida, 12 mg/m2 or dnr, 60 mg/m2 d1-2 and AraC 3 g/m2/12h d1-4) then autologous HSCT (auto-HSCT) prepared with busulfan (4 mg/kg, d-6 to d-3) and melphalan (140 mg/m2, d-2) or two courses of high-dose AraC (HiDAC). Since 2008, patients with intermediate-risk cytogenetic and favorable genotype (mutation of *NPM1* or *CEBPA* without *FLT3-ITD* mutation) were no longer allocated to allo-HSCT in first CR and mainly received three cycles of HiDAC as consolidation. The induction chemotherapy for patients older than 60 years (n=38) combined ida (8 mg/m2, d1-5), AraC (100 mg/m2, d1-7) with (n=17) or without (n=21) lomustine (200 mg/m2, d1). Patients achieving CR received a consolidation with ida (8 mg/m2, d1-3) and AraC (50 mg/m2/12h, d1-5) then maintenance therapy with ida at d1 only and the same scheme of AraC. Bone marrow AML samples were obtained after informed consent in accordance with the Declaration of Helsinki. All samples were stored in the HIMIP tumor bank of the U1037 Inserm department (n°DC-2008-307-CPTP1 HIMIP). The study was approved by the institutional review board.

### Mutation screening

The mutational status of *DNMT3A* exon 23 along with 3 additional mutations (*FLT3 TKD* exon 20, *IDH1* and *IDH2* exons 4) were analyzed by high-resolution melting (HRM) PCR using the LightCycler 480 with High Melting Resolution Master Mix 1X (Roche Applied Science), with 10 ng genomic DNA, 0.1 μmol/l of each primer (see below), and 25 mmol/l MgCl2. HRM PCR cycling conditions were initial denaturation at 95°C for 10 minutes, followed by 50 cycles at 95°C for 10 seconds, 50 cycles at 63°C for 15 seconds, and 50 cycles at 72°C for 25 seconds. Melting curve was measured from 72°C to 95°C, with 25 acquisitions per degree centigrade. Primer sequences were as follows: DNMT3A_X23_F1, 5′-CTG GCC AGC ACT CAC CCT-3′; DNMT3A_X23_R1, 5′-TGT TTA ACT TTG TGT CGC TAC CTC A-3′; FLT3_X20_F2, 5′-TCA CAG AGA CCT GGC CGC-3′; FLT3_X20_R1, 5′-TGC CCC TGA CAA CAT AGT TGG-3′; IDH1_X4_F1, 5′-GGC TTG TGA GTG GAT GGG TAA-3′; IDH1_X4_R2, 5′-GCA TTT CTC AAT TTC ATA CCT TGC TTA-3′; IDH2_X4_R140_F1, 5′-GAA AGA TGG CGG CTG CAG T-3′; IDH2_X4_R140_R3, 5′-TGT TTT TGC AGA TGA TGG GC-3′; IDH2_X4_R172_F4, 5′-GAT GTG GAA AAG TCC CAA TGG A-3′; IDH2_X4_R172_R2, 5′-CAC CCT GGC CTA CCT GGT C-3′. Positive cases detected by the HRM analysis were sequenced to confirm the mutation. FLT3 exon 13 ITD and *NPM1* mutation screening were performed using a multiplex PCR using Gold Taq DNA polymerase (Applied Biosystems) and the following primers: HsNPM1_X12_F1, 5′-GAA GTG TTG TGG TTC CTT AAC-3′; HsNPM1_X12_R1FAM, 5′(FAM)-TGG ACA ACA CAT TCT TGG CA-3′; FLT3_NEM_E, 5′-TGG TGT TTG TCT CCT CTT CAT TGT-3′; and FLT3_NEM_Qned, 5′(NED)-GTT GCG TTC ATC ACT TTT CCA A-3′. PCR products were analyzed on a sequencer using sizing fragment analysis. *CEBPA* screening was performed according to Pabst et al [13].

### NOD/SCID mice xenograft

Adult NOD/SCID mice (6-8 weeks old) were sublethally irradiated with 250 cGy of total body irradiation 24 h before injection of leukemic cells. Leukemia samples were thawed at room temperature, washed twice and suspended in PBS at a final concentration of 1-2 million cells per 200 μL of PBS per mouse for IV injection. Daily monitoring of mice for symptoms of disease (ruffled coat, hunched back, weakness, or reduced motility) determined the time of killing for injected animals with signs of distress. If no sign of distress was seen, mice were analyzed 12 weeks after injection except as otherwise noted. For assessment of leukemic engraftment, NOD/SCID mice were humanely killed in accordance with IACUC protocols. Bone marrow (mixed from tibias and femurs) and spleen were dissected in a sterile environment, flushed in PBS and made into single cell suspensions for analysis by flow cytometry (FACS Calibur, BD Biosciences, San Jose, CA USA).

### Statistical analysis

Comparisons of patient characteristics (covariates) were performed using the Mann-Whitney test for continuous variables and the Fisher's exact test for categorical variables. Complete remission (CR) was defined following Cheson criteria [14]. In univariate analysis, covariates associated with response to induction therapy or outcome were identified using Fisher's exact test then included in a multivariable logistic model. Overall survival (OS) and disease-free survival (DFS) rates were measured from the date of diagnosis until death and from the date of CR until death or relapse, respectively. Patients alive were censored at the time of last contact. OS and DFS were estimated by the Kaplan-Meier method and compared using the log-rank test. All calculations were performed using GraphPad Prism software, version 5.0 (GraphPad Software Inc., La Jolla, CA). Survival-time data (DFS and OS) and covariates were analyzed using the backward method of Cox proportional hazards regression.

## RESULTS

### *DNMT3A* exon 23 mutation is a frequent event in adult AML

*DNMT3A* exon 23 screening was performed on available samples coming from 288 AML patients aged from 18 to 65-year old and treated in Toulouse between 2000 and 2009. *DNMT3A* exon 23 mutations were detected in 39 patients (13.5%) occurring almost exclusively at the R882 codon (23 R882H; 13 R882C; 2 R882P) and in one patient at the W893 codon (W893S). No association between *DNMT3A* exon 23 mutations and age or sex was detected.

### *DNMT3A* exon 23 mutations occur exclusively in intermediate cytogenetic risk group

Patients were cytogenetically subdivided according to the MRC 2010 classification resulting in 46 patients with low risk, 194 with intermediate risk and 43 with high risk whereas five patients were not classified in absence of karyotypes [15]. Patients with a *DNMT3A* exon 23 mutation were exclusively identified in the intermediate-risk group (39/194, 20%) as compared to CBF and high-risk AML (p<0.001), 27 with a normal karyotype and 12 with various associated abnormalities, the most frequent being an additional copy of the chromosome 8 (3 patients) or 13 (2 patients). No *DNMT3A* exon 23 mutation was detected in high or low risk groups. Consequently, we focused the analysis exclusively on the 194 patients with intermediate-risk.

### *DNMT3A* exon 23 mutations are associated with FAB M4/M5 subtypes and a higher WBC count

The characteristics of intermediate-risk patients according to *DNMT3A* mutational status are listed in table 1. A bias toward monocytic differentiation (FAB groups M4 and M5) was identified in *DNMT3A* exon 23+ samples (p<0.0001). A strong association was found between *DNMT3A* exon 23+ and a higher white blood cell count (p=0.001). Platelet count, hemoglobin concentration and marrow blast percentage at diagnosis were similar in *DNMT3A* exon 23+ and – groups.

**Table 1:** Characteristics of AML patients with intermediate-risk according to *DNMT3A* exon 23 mutation

	No DNMT3A exon 23 mutation	DNMT3A exon 23 mutation	p
**Sex - no.**			
** Male**	78	17	0.48
** Female**	77	22	
**Age - y**			
** Median**	53	47	0.051
** Range**	18-65	20-63	
**WBC - G/L**			
** Median**	14.6	52	<0.0001
** Range**	0.8-356	1.0-250	
**Hb - g/dL**			
** Median**	9.6	10.4	0.22
** Range**	4.3-16	4.8-13.2	
**Platelets - G/L**			
** Median**	70	62	0.54
** Range**	5-964	8-814	
**FAB AML subtypes - no.**			
** M0/M1/M2**	4/43/45	0/3/6	<0.0001
** M4 /M5**	26/27	17/11	
** M6/M7**	0/0	0/0	
** Unclassified**	10	1	
**Normal Karyotype no. (%)**	96 (62)	27 (69)	0.46
**Mutations - no. /total no. (%)**			
** FLT3-ITD**	37/155 (24)	17/39 (44)	0.027
** FLT3-TKD**	3/62 (5)	1/14 (7)	0.57
** NPM1**	49/155 (32)	29/39 (74)	<0.0001
** CEBPA**	20/155 (13)	2/39 (5)	0.26
** IDH1**	16/155 (10)	8/39 (21)	0.10
** IDH2**	16/155 (10)	5/39 (13)	0.77
** KIT exon 17**	2/155 (1)	0/39 (0)	-
**Complete response - no. (%)**	126/155 (81)	34/39 (87)	0.48
**Allogeneic SCT - no./total no. (%)**	40/155 (26)	16/39 (41)	0.075
**Autologous SCT - no./total no. (%)**	28/155 (18)	7/39 (18)	1.00
**Relapse - no./total no. (%)**	63/126 (50)	14/34 (41)	0.36
**5-year survival (%)**	36.5	47.3	-

**WBC, white blood cell count; FAB, French American British; SCT, stem-cell transplantation.**

### *DNMT3A* exon 23 mutations are associated with NPM1 and FLT3-ITD mutations

*DNMT3A* exon 23 mutations were significantly associated with *FLT3*-ITD (17 *FLT3-ITD*/39 *DNMT3A* exon 23+, 44% vs. 37/155 *DNMT3A* exon 23-, 24%, p=0.027), *NPM1* mutations (29 *NPM1c*/39 *DNMT3A* exon 23+, 74% vs. 49/155 *DNMT3A* exon 23-, 32%, p< 0.001). No relationship was identified between *DNMT3A* exon 23 mutations and *CEBPA* mutations, *IDH1 R132, IDH2 R140* and *R172* mutations, *FLT3* tyrosine kinase domain or *KIT* exon 17 mutations. The significant relationship between *DNMT3A* exon 23 mutations and *NPM1* mutations was independent of the *FLT3-ITD* mutation status (p<0.001 in *FLT3-ITD+* or *FLT3-ITD-* in both groups). In contrast, the relationship between *DNMT3A* exon 23 mutations and *FLT3-ITD* mutations was lost in the *NPM1* mutation subgroups (p=0.700 in *NPM1c -* and p=0.043 in *NPM1c* + patients), suggesting that the relationship between *DNMT3A* and *NPM1* mutations was stronger than the relationship between *DNMT3A* and *FLT3-ITD* mutations.

### *DNMT3A* exon 23 mutations are conserved in xenograft mice models

We next analyzed the mutational status of nine primary AML samples which showed engraftment capacities in xenograft NOD/SCID mice model. The presence of *FLT3-ITD* (seven out of nine, data not shown) correlated with the ability to engraft in these mice as it has been shown earlier [16-18]. Furthermore, we have also observed that four of these nine specimens carried the *DNMT3A* exon 23+ mutations that were associated with *FLT3-ITD* and *NPM1* mutations (Table 2). For these four samples, we also analyzed their post-transplantation mutational status and found that *DNMT3A* exon 23+ mutations were conserved in NOD/SCID mice. Overall, these data show a stability of the *DNMT3A* mutations in AML engrafted mice and suggest a preferential engraftment of primary AML specimens with triple *FLT3-ITD/NPM1/DNMT3A* mutations in immunodeficient mice.

**Table 2:** Distribution of *FLT3-ITD, NPM1* and *DNMT3A* exon 23 mutations in AML samples sorted from NOD/SCID mice

Patient#	Tx status	Engraftment	Gender	FAB	Karyotype	Status	DNMT3A	FLT3	NPM1	IDH1	IDH2	IDH2	CEBPA	KIT
		mean%				Dx/Rel				R132	R140	R172		
**LAM018**	Pre		M	5	normal	Dx	+	ITD	+	WT	WT	WT	WT	WT
	**Post**	**83**					**+**	**ITD**	**+**	**WT**	**WT**	**WT**	**WT**	**WT**
**LAM016**	Pre		M	5	normal	Rel	+	ITD	WT	WT	WT	WT	WT	WT
	**Post**	**81**					**+**	**ITD**	**WT**	**WT**	**WT**	**WT**	**WT**	**WT**
**LAM002**	Pre		M	1	normal	Dx	+	ITD	+	WT	WT	WT	WT	WT
	**Post**	**38**					**+**	**ITD**	**+**	**WT**	**WT**	**WT**	**WT**	**WT**
**LAM007**	Pre		F	4	normal	Dx	+	ITD	+	WT	WT	WT	WT	WT
	**Post**	**7**					**+**	**ITD**	**+**	**WT**	**WT**	**WT**	**WT**	**WT**

**Tx, transplantation; FAB, French American British; Dx, diagnosis; Rel, relapse; WT, wild type; ND, not done.**

### *DNMT3A* exon 23 mutations have no prognostic impact in adult AML with intermediate-risk cytogenetics

**Figure 2:**
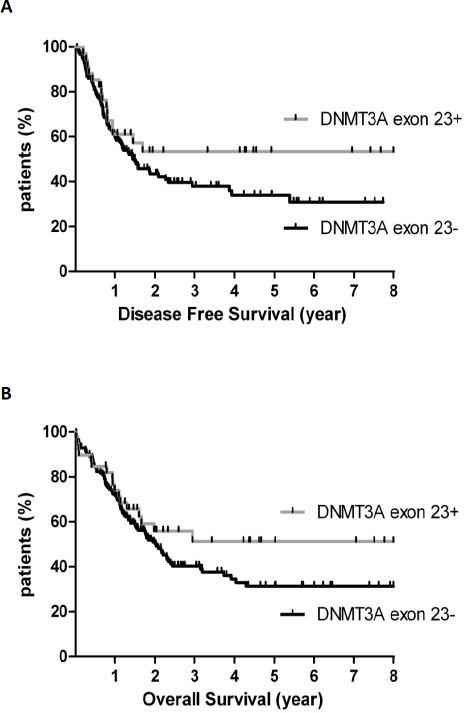
Prognostic impact of *DNMT3A* exon 23 mutations in 194 AML patients with intermediate-cytogenetic risk (A) Disease-free (DFS) and (B) overall survival (OS) in patients with or without *DNMT3A* exon 23 mutations. DFS and OS were not different between DNMT3A exon 23+ and *DNMT3A* exon 23- patients (p=0.16 and p=0.17, respectively).

Analysis of the therapeutic outcome was performed for the 194 intermediate-risk patients. Of these patients, 160/194 (83%) achieved CR after induction chemotherapy. The CR rate did not differ according to *DNMT3A* exon 23 mutational status, with 34/39 (87%) and 126/155 (81%) CR in the *DNMT3A* exon 23+ group and *DNMT3A* exon 23- group, respectively (p=0.48). CR rate was significantly influenced by leukocytosis with a cut-off at WBC>30 G/L (95% CI, 0.14-0.68, OR, 0.3, p=0.01) but not by age, *CEBPA, NPM1* or *FLT3-ITD* mutations. In the *DNMT3A* exon 23+ group, 16 (41%) and 7 (18%) patients in first CR received allo-SCT or auto-SCT as consolidation therapy, not significantly different from the *DNMT3A* exon 23- group in which 40 (26%) and 28 (18%) received allo-SCT or auto-SCT, respectively. In these complete responders, 15 events were observed in the *DNMT3A* exon 23+ group and 72 events in the *DNMT3A* exon 23- group (44% and 57% of CR patients, respectively). Disease-free survival was not significantly different between *DNMT3A* exon 23+ (median not reached) and *DNMT3A* exon 23- patients (median DFS, 17.6 months) (95% CI, 0.87-2.33, HR 1.42, p=0.16) (figure 2A). There were 17 deaths (44%) in the *DNMT3A* exon 23+ group and 88 (57%) in the *DNMT3A* exon 23- group. Overall survival was not significantly different between *DNMT3A* exon 23+ (median not reached) and *DNMT3A* exon 23- patients (median OS, 24.7 months) (95% CI, 0.87-2.19, HR 1.38, p=0.17) (figure 2B). Among age, WBC count, *DNMT3A* exon 23 mutations and *FLT3/NPM1* genotypes, the only factor that significantly influenced both DFS and OS was the *FLT3wt*/*NPM1c* genotype (not shown).

### AML patients with *DNMT3A* exon 23 mutations may benefit from idarubicin as compared to daunorubicin

**Figure 3:**
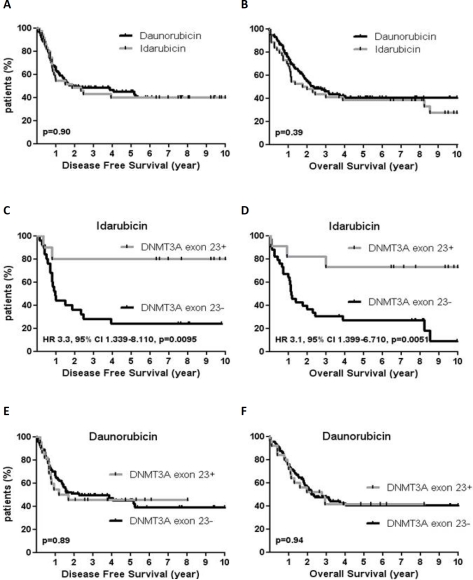
Impact of idarubicin in 156 AML patients ≤ 60y according to *DNMT3A* exon 23 mutations DFS (A) and OS (B) according to daunorubicin or idarubicin treatment. DFS (C) and OS (D) in patients treated by idarubicin according to *DNMT3A* exon 23 mutations. DFS (E) and OS (F) in patients treated by daunorubicin according to *DNMT3A* exon 23 mutations.

**Figure 4:**
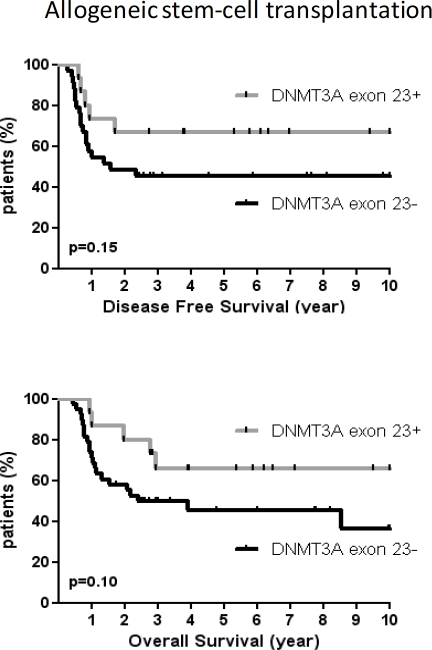
Outcome of allografted patients according to *DNMT3A* exon 23 mutations DFS (A) and OS (B) in DNMT3A exon 23+ patients (n = 15) and *DNMT3A* exon 23- patients (n=38) who were allografted in first complete response.

As we did not find any prognostic impact of *DNMT3A* exon 23+ mutations in contrast to previous studies, we tried to find out whether treatments received by *DNMT3A* exons 23+ patients could explain these differences.[9;19-20] Because patients who were older than 60 received idarubicin and lomustin but also because they were much less frequently allografted, we focalized our analysis on younger patients (60y or less). The characteristics of patients according to the type of anthracycline used at induction are reported in table 3. There was no difference between patients treated by daunorubicin and those treated by idarubicin in terms of DFS (median DFS, 22.7 months for DNR *vs* 22.8 months for Ida, p=0.90) and OS (median OS, 28.5 months for DNR *vs* 24.4 months for Ida, p=0.39). Analysis of covariates associated with both DFS and OS are shown in table S1-S2. However, there was a significant impact of *DNMT3A* exon 23 mutations in patients treated with idarubicin. *DNMT3A* exons 23+ patients had better DFS (not reached *vs* 11.6 months, HR 3.3, 95% CI, 1.34-8.11, p=0.009) and OS (not reached *vs* 14.3 months, HR 3.1, 95% CI, 1.39-6.71, p=0.005) as compared to *DNMT3A* exons 23- patients when treated with idarubicin whereas patients treated with daunorubicin had similar outcome regardless the DNMT3A mutational status (figure 3). Conversely, the outcome of patients with *NPM1+/FLT3wt* genotype was not impacted by the type of anthracycline used in induction (Figure S1). In patients who received allo-SCT (n=53), there was a trend for improved outcome in *DNMT3A* exon 23+ patients (DFS and OS medians not reached) as compared to *DNMT3A* exon 23- patients (median DFS, 19.1 months, p=0.15; median OS, 29.2 months, p=0.1) but the difference did not reach statistical significance (figure 4). To better address the effects of idarubicin *vs*. daunorubicin along with other variables in *DNMT3A* mutated patients, we performed univariate and multivariate analysis. As shown in table 4, idarubicin had an independent favorable prognostic effect on OS (HR 0.27, 95% CI, 0.08-0.97, p=0.046) when considering age, karyotype, *NPM1/FLT3wt* genotype, WBC and allo-SCT in *DNMT3A* mutated patients.

**Table 3:** Characteristics of patients with intermediate-cytogenetic risk according to the type of anthracycline used at induction treatment

	No DNMT3A exon 23 mutation		DNMT3A exon 23 mutation		p
	DNR	IDA	DNR	IDA	
**Sex - no.**					
** Male**	39	20	8	6	0.16
** Female**	48	13	17	5	
**Age - y**					
** Median**	49	50	47	46	0.81
** Range**	21-60	18-60	20-60	35-60	
**WBC - G/L**					
** Median**	14.5	17.9	52	41.3	0.0037
** Range**	0.8-356	1.8-220	1.7-249	1.0-250	
**Mutations - no. /total no. (%)**					
** FLT3-ITD**	20/87 (23)	11/33 (33)	11/25 (44)	4/11 (36)	0.19
** NPM1**	30/87 (34)	5/33 (15)	19/25 (76)	8/11 (73)	<0.0001
** CEBPA**	12/87 (13)	6/33 (18)	2/25 (8)	0/11 (0)	0.38
** IDH1**	11/87 (13)	2/33 (6)	6/25 (24)	2/11 (18)	0.24
** IDH2**	10/87 (11)	2/33 (6)	3/25 (12)	1/11 (9)	0.83
**Complete response - no. (%)**	73/87 (84)	25/33 (76)	22/25 (88)	10/11 (91)	0.53
**Allogeneic SCT - no./total no. (%)**	27/73 (36)	11/25 (44)	10/22 (45)	5/10 (50)	0.71
**Relapse - no./total no. (%)**	32/73 (44)	17/25 (68)	12/22 (55)	1/10 (10)	0.014
**5-year survival (%)**	40.1	26.9	41.4	72.7	-

**DNR, daunorubicin; IDA, idarubicin; WBC, white blood cell count; SCT, stem cell transplantation.**

**Table 4:** Univariate and Multivariate Analysis for DFS and OS in *DNMT3A* exon 23 mutated patients Analysis of covariates associated with DFS and OS. P of the univariate analysis is the p value of the Log rank test. HR is the value of the hazard ratio. 95% CI is the 95% confident interval of the hazard ratio. Data of AML patients with *DNMT3A* exon 23 mutations were complete and were included in the Cox proportional-hazards regression. *NPM1*: Nucleophosmin; *FLT3-ITD*: internal tandem duplication of the *FLT3* gene; Allo-SCT: Allogeneic stem-cell transplantation; Ida: idarubicin; WBC: white blood cell count.

	Univariate analysis			Multivariate analysis		
**DFS**	p	HR	95% CI	p	HR	95% CI
** Age > 50y**	0.94	1.04	0.35-3.09	>0.1		
** NPM1+/FLT3-ITD-**	0.49	0.70	0.25-1.95	>0.1		
** Normal karyotype**	0.95	1.04	0.33-3.24	>0.1		
** Allo-SCT**	0.13	0.45	0.16-1.26	>0.1		
** Ida treatment**	0.036	0.32	0.11-0.93	0.052	0.21	0.05-1.01
** WBC count > 30G/L**	0.55	1.43	0.45-4.56	>0.1		
**OS**						
** Age > 50y**	0.76	1.17	0.43-3.14	>0.1		
** NPM1+/FLT3-ITD-**	0.52	0.74	0.29-1.89	>0.1		
** Normal karyotype**	0.88	1.08	0.39-2.99	>0.1		
** Allo-SCT**	0.06	0.41	0.16-1.04	0.053	0.36	0.12-1.01
** Ida treatment**	0.046	0.37	0.14-0.98	0.046	0.27	0.08-0.97
** WBC count > 30G/L**	0.2	2.06	0.69-6.13	>0.1		

## DISCUSSION

In accordance with the first study assessing *DNMT3A* mutations in AML, we confirm here the high prevalence of *DNMT3A* exon 23 mutations in a series of 288 *de novo* adults AML [9]. We have also observed that these mutations were exclusively found within the intermediate-risk cytogenetic group, associated with M4/M5 FAB subtypes and hyperleukocytosis. At the molecular level, *DNMT3A* exon 23 mutations were strongly associated with *NPM1* mutations and to a lesser extent with *FLT3-ITD* mutations whereas no correlation was found with *CEBPA, KIT* or *IDH1/2* mutations.

However, in contrast with previous studies, we failed to show any prognostic impact of *DNMT3A* exon 23 mutations in AML with intermediate-risk cytogenetic [9;19-20]. We acknowledge that our patients had been treated over a long period of time (nine years) and this could have potentially introduced some bias. Indeed, two major changes in the management of AML patients had been undertaken in our center during this period of time: the introduction of primary prophylaxis of invasive fungal infections using azoles (voriconazole from 2003 to 2008, posaconazole thereafter) [21] and the stratification of allo-HSCT indication according to the new molecular classification of intermediate-risk AML using *CEBPA, FLT3 and NPM1* mutations. Indeed, patients with wild type *FLT3* and *CEBPA* or *NPM1* mutations were no longer allocated to allo-HSCT in first CR since the results published by Schlenk *et al*.[5]. However, since these two measures were applied in 2003 and 2008 respectively, their impact on outcome, if any, could have been negligible in the whole cohort of patients. Furthermore, our induction and consolidation regimen have little changed for fifteen years, particularly the dosing of anthracyclines, with daunorubicin always given at 60 mg/m^2^/day for three days at time of the induction chemotherapy. By contrast, the doses of anthracyclines are not fully described in the study of Ley et al, in which several induction regimen were used, some of them using the infra optimal dose of 45 mg/m^2^/d [22-24]. Moreover, some patients received hypomethylating agents or lenalidomide which generally induce fewer responses than intensive chemotherapy in AML patients [25-26]. Also, complete response and early death rates were not mentioned and this could be of interest as patients with R882 *DNMT3A* mutations usually have a high white blood cell count, a recognized risk factor for early death. In our study, *DNMT3A* exon 23 mutations did not impact on both complete response and early death rates. Moreover, we found that patients with *DNMT3A* exon 23 mutations could specifically benefit from idarubicin although the small number of patients in our study requires confirmation in larger cohorts. The strong impact of *DNMT3A* mutations on the response to idarubicin as compared to daunorubicin is quite unexpected. However, it has been recently shown that DNMT3A could play a role in anthracyclines-induced apoptosis of colorectal cancer cells. Indeed, the expression of DNMT3A is upregulated at apoptosis-inducing concentrations of doxorubicin and involved in p21 repression thereby blocking senescence [27]. Whether this program is induced by idarubicin but not daunorubicin in DNMT3A mutated/haploinsufficient cells remains to be determined. Alternatively, *DNMT3A* mutations could specifically impact on the expression of genes involved in idarubicin metabolism as compared to daunorubicin. The broader spectrum of activity of idarubicin has been attributed to increased lipophilicity, cellular uptake and improved stabilization of a ternary drug-topoisomerase II-DNA complex [28]. Thus, the discrepancy in therapeutic outcome between our series and those previously described could be due to differences in treatment intensity or altered metabolism of anthracyclines. Several clinical trials have already assessed the impact of daunorubicin dose intensification or compared idarubicin *vs*. daunorubicin in large cohorts of AML patients. Reassessing results of these controlled studies in light of *DNMT3A* mutational status could easily confirm or not our preliminary observations [12;22-23;29-30]. It remains also to be determined if DNMT3A mutations affect in a similar way the metabolism of other compounds that intercalates DNA and inhibits topoisomerase II such as the new quinolone derivative vosaroxin which is currently assessed in AML [31].

In addition, we were also able to demonstrate for the first time that *DNMT3A* mutations are also found in leukemic samples engrafted in immunodeficient mice. Although we could not demonstrate that the *DNMT3A* exon 23 mutation is a surrogate marker of engraftment in NOD/SCID mice, it should be noted that all mutated samples tested recapitulated the initial disease in mice. By comparison, it has been shown that only 50% of intermediate-risk cytogenetic samples display engraftment properties in NOD/SCID mice [32]. This suggests that engraftment properties of AML samples should be assessed in light of specific molecular lesions. It has been shown that engraftment in xenograft NOD/SCID-IL2Rγc^−/−^ mice model did not correlate with French-American-British subtype or cytogenetic abnormalities but with the presence of *FLT3*-*ITD* [17]. More recently, Sarry et al. have observed that a high engraftment level occurred within human primary AML samples carrying at least two mutations (seven out of nine samples had *FLT3-ITD* and *NPM1* mutations) and that these mutations were strongly conserved in the different leukemic stem cell populations sorted as well as still present after the first transplantation into NOD/SCID-IL2Rγc^−/−^ mice [18]. In the present study, we show that the *DNMT3A* mutation is also conserved through the NOD/SCID xenograft model and is associated with higher leukemic engraftment level in cooperation with *FLT3-ITD* and *NPM1* mutations, suggesting that this mutation occurs in the early leukemogenic events and belongs to the main leukemic clone over the course of the disease progression.

In summary, our data confirm that *DNMT3A* exon 23 mutations can be easily evaluated in medical practice and represent a frequent molecular event in intermediate-risk AML associated with *NPM1* and *FLT3-ITD* mutations but have no clear impact on disease-free and overall survival. However, although this finding needs to be confirmed in largest cohort of patients, *DNMT3A* mutations could be a predictive factor for response to idarubicin and, thus could have a direct influence in the way AML patients should be managed.

## Supplementary Figure and Tables




