# Supplementary figures and images for: Do AML patients with DNMT3A exon 23 mutations benefit from idarubicin as compared to daunorubicin? A single center experience
Source: Oncotarget. 2011 Nov 9;2(11):850–61. doi: 10.18632/oncotarget.347 (PMC3260002; doi:10.18632/oncotarget.347)

**A**

Figure S1

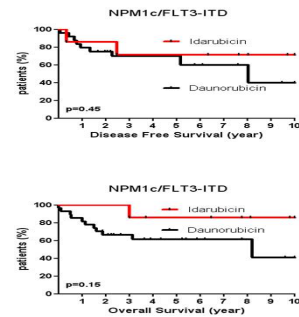

**B**

Figure S1

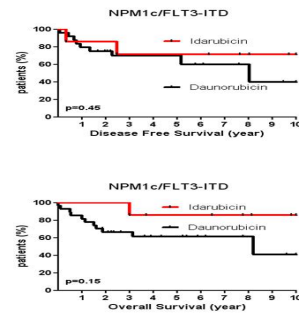

Figure S1: Survival Curves

Supplement: Supplementary file 1 [file oncotarget-02-850-s001.pdf]
