# Supplementary material for: Do AML patients with DNMT3A exon 23 mutations benefit from idarubicin as compared to daunorubicin? A single center experience
Source: Oncotarget. 2011 Nov 9;2(11):850–61. doi: 10.18632/oncotarget.347 (PMC3260002; doi:10.18632/oncotarget.347)
Supplement: Supplementary file 2 [file oncotarget-02-850-s002.pdf]

Table S1. Analysis of covariates associated with the disease free survival. P of the univariate analysis is the p value of the Log rank test. HR is the value of the hazard ratio. 95% CI is the 95% confident interval of the hazard ratio. Data of 130 patients were complete and were included in the Cox proportional-hazards regression.

DFS	Univariate analysis			Cox regression		
	**p**	**HR**	**95% CI**	**p**	**HR**	**95% CI**
**Age > 50y**	0.86	1.04	0.66-1.64	>0.1		
**NPM+ FLT3-ITD-**	0.005	0.49	0.30-0.81	0.001	0.36	0.34-0.84
**normal karyotype**	0.42	0.82	0.51-1.33	>0.1		
**allograft**	0.17	0.73	0.46-1.15	0.006	0.53	0.19-0.65
**IDA treatment**	0.99	1.00	0.60-1.67	>0.1		
**WBC count > 30G/L**	0.68	1.10	0.70-1.75	>0.1		
**DNMT3a mutation**	0.22	0.72	0.43-1.22	>0.1		

Table S2. Analysis of covariates associated with the overall survival. P of the univariate analysis is the p value of the Log rank test. HR is the value of the hazard ratio. 95% CI is the 95% confident interval of the hazard ratio. Data of 156 patients were complete and were included in the Cox proportional-hazards regression.

OS	Univariate analysis			Cox regression		
	**p**	**HR**	**95% CI**	**p**	**HR**	**95% CI**
**Age > 50y**	0.81	1.05	0.70-1.59	>0.1		
**NPM+ FLT3-ITD-**	0.003	0.49	0.31-0.79	0.003	0.37	0.19-0.71
**normal karyotype**	0.24	0.77	0.50-1.19	>0.1		
**allograft**	0.04	0.64	0.42-0.98	0.049	0.61	0.37-0.99
**IDA treatment**	0.46	1.19	0.75-1.87	>0.1		
**WBC count > 30G/L**	0.19	1.32	0.87-1.99	>0.1		
**DNMT3a mutation**	0.19	0.73	0.46-1.17	>0.1		
